# An Absent Lingual Frenulum in a Non-syndromic Premature Infant

**DOI:** 10.7759/cureus.40402

**Published:** 2023-06-14

**Authors:** Abduljabbar A Alyamani, Nasser Almutairi, Waleed A Alshareef, Latifa AlMakoshi

**Affiliations:** 1 Otolaryngology - Head and Neck Surgery, King Faisal Specialist Hospital and Research Centre, Riyadh, SAU; 2 Otolaryngology - Head and Neck Surgery, King Saud University Medical City, Riyadh, SAU

**Keywords:** direct laryngobronchoscopy, swallowing assessment, abnormal frena, ehler’s danlos syndrome, premature infant, non-syndromic, lingual frenulum

## Abstract

The lingual frenulum (LF) is a fold of tissue that connects the tongue to the oral cavity’s floor. Abnormal frenula are associated with speech alterations. The absence of the LF is associated with Ehler’s Danlos syndrome (EDS). In this case report, we present a premature infant incidentally found to have an absent lingual frenulum, with recurrent desaturations during feeding. The desaturations were believed to be due to the absent lingual frenulum, but they resolved after one month without treatment and were then attributed to apnea of prematurity. Whole exome sequence showed no genetic disorders. The infant is now doing well with no interventions. An absent lingual frenulum warrants molecular genetic testing for EDS. However, it does not warrant any treatment; special considerations are only required during intubation.

## Introduction

A frenulum is a small fold of integument or mucous membrane that checks, curbs, or limits the movements of an organ or part [1]. Several frenula are usually present in a normal oral cavity, the most notable among them being the maxillary labial frenum, mandibular labial frenum, and lingual frenum (LF) [2]. The LF is a midline fold of tissue that connects the tongue’s ventral surface to the oral cavity’s floor [3]. A normal LF has an insertion from the midline under the tongue to the floor of the mouth beneath the inferior alveolar ridge. There are many anatomical variations of the LF, including a frenulum with anterior insertion, a short frenulum, and a short frenulum with anterior insertion. Abnormal frenula can lead to speech alterations [4]. In some situations, the LF may be absent. This absence is associated with Ehlers-Danlos syndrome (EDS). In a study of 40 patients with EDS, 33 patients had an absent LF, and two had LF hypoplasia [5]. A previous case report noted a nonsyndromic female patient with an absent LF in Saudi Arabia discovered incidentally during a dental visit. She did not have a speech impediment and did not meet the diagnostic criteria for EDS. A family screening indicated neither a family history of EDS nor an absent LF [6]. Similarly, the current case report presents a nonsyndromic premature infant who was found to have an absent LF. A review of the relevant literature is also presented.

## Case presentation

**Figure 1:**
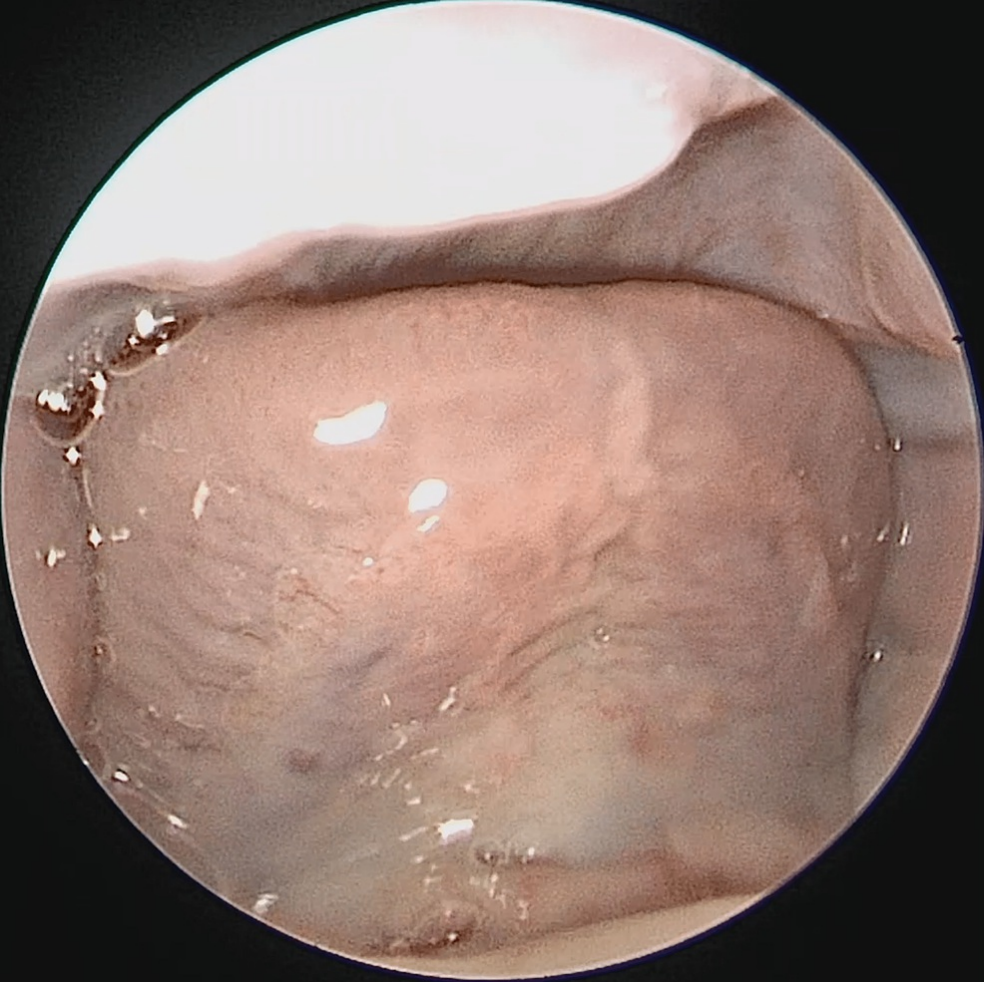
Anterior view of the ventrum of the tongue showing an absent frenulum

A baby boy was born prematurely at 34 weeks. The delivery was uneventful, with Apgar scores of 8 and 9 at one and five minutes, respectively. He spent one week in the neonatal intensive care unit (NICU) after delivery due to prematurity. During the general examination, an absent LF was noticed because his tongue tip was observed to be consistently touching the hard palate. The pediatric otolaryngology team was consulted for further evaluation due to desaturations as severe as 50% during feeding since birth and an absent LF. There was no history of choking, nasal regurgitation, shortness of breath, or stridor. He had no family history of EDS, and his mother had no history of travel to an area where the Zika virus is endemic. On physical examination, the baby was not dysmorphic, and an oral cavity examination was normal apart from an absent lingual frenulum (Figure 1). The examination of the neck, nose, and ears was also unremarkable. Bedside swallowing assessment showed good oral and oropharyngeal phases of swallowing with no signs of aspiration. Cranial ultrasound and echocardiography were normal.

At seven weeks, the patient underwent direct laryngobronchoscopy (DLB), which showed mild right-sided tracheomalacia throughout the length of the trachea. There were no laryngeal clefts or tracheoesophageal fistulae. The patient underwent genetic testing for EDS, which was normal. The genetic testing of the whole exome sequence showed no genetic disorders.

One week after the DLB, desaturation events stopped spontaneously. It seems that these desaturations were due to apnea of prematurity. The patient was discharged home and outpatient clinic follow-up was advised. After one month, the patient no longer showed any desaturations and his tongue was also in its natural position despite the absent LF. No further management was deemed necessary.

## Discussion

The development of the tongue begins in the fourth week of gestation. It consists of contributions from the first four pharyngeal arches. The anterior two-thirds of the tongue are formed by the first arch and receive sensory innervation from the trigeminal nerve. The posterior third comprises contributions from the second through fourth pharyngeal arches and receives sensory innervation from the glossopharyngeal nerve [7]. In the sixth week of gestation, as the tongue develops, the frenum forming cells undergo apoptosis, shrinking away from the tip, resulting in the final range of mobility of the tongue [8]. The tongue’s musculature is divided into intrinsic and extrinsic muscles. The hypoglossal nerve supplies all intrinsic and extrinsic muscles except for the palatoglossal muscle, which is supplied by the vagus nerve [9]. The LF is a midline fold of tissue connecting the ventral surface of the tongue to the floor of the oral cavity [3]. Although it was previously believed that the LF is a submucosal string or band, recent studies have shown that it is a dynamic structure formed by a fold in the floor of the mouth fascia [10].

A short LF has been implicated in ankyloglossia. Ankyloglossia, also known as tongue tie, is a condition in which the LF limits tongue movement, leading to feeding problems during infancy and speech. However, there is still no standard definition of it [11]. The prevalence of ankyloglossia among infants is around 8% [12]. Although ankyloglossia is due to a short LF, no anatomical variants of the LF have been found to correlate directly with impaired tongue mobility [10].

The absence of the LF is a far less common condition and was associated with congenital Zika syndrome (CZS) and EDS [5,13]. While no study has assessed its overall prevalence, a study of 40 EDS patients conducted in Italy assessing the absence of LF among EDS patients found that it was absent in 33 patients and hypoplastic in three of them, and an overall 90% sensitivity of an absent or hypoplastic LF for EDS was indicated [5]. A French study including 43 patients and 86 controls showed that an absent LF had 53.5% sensitivity and 98% specificity for EDS and was most common in patients with vascular EDS [14]. Initially, it was believed that CZS could cause an absent LF. However, a study in Brazil that assessed 54 patients with CZS found that what was believed to be an absent frenulum in 37% of the patients was actually a posteriorly positioned frenulum or a submucous frenulum [13].

Although an absent LF does not warrant any management, it can present a problem during intubation due to increased tongue mobility and subsequent susceptibility to injury. This problem can be circumvented by suturing the tip of the tongue or using other maneuvers to stabilize it [15].

## Conclusions

An absent LF is a relatively rare condition that is often discovered incidentally and is associated with EDS. However, it can be found in non-syndromic patients. Thorough clinical examination, with a particular focus on the oral cavity and genetic testing, are critical when suspecting EDS. The isolated absence of the LF does not warrant any specific management apart from special consideration during intubation. 
